# Opinion Dynamic and Social Clustering in a 2D Space: An Agent Based Experiment

**DOI:** 10.1007/s10614-025-10961-w

**Published:** 2025-05-21

**Authors:** Francesco Pasimeni, Robert Wade, Floor Alkemade

**Affiliations:** Technology, Innovation and Society Research Group, Department of Industrial Engineering and Innovation Sciences, Eindhoven University of Technology, Atlas 5.402, P.O. Box 513, 5600 MB Eindhoven, The Netherlands

**Keywords:** Opinion dynamics, Polarisation, Opinion clusters, Attraction-repulsion model

## Abstract

This paper studies the impact of movement and interactions of individuals on opinion dynamics and polarisation within a two-dimensional (2D) space. By extending the Attraction-Repulsion Model (ARM), we explore how agents form opinion clusters based on geographic constraints and social interactions. Our model illustrates that individuals with similar opinions tend to form like-minded groups, with higher visibility leading to fewer, larger clusters. Conversely, low tolerance drives high polarisation. Geographic constraints result in larger clusters centralising to avoid conflicts, while smaller clusters remain peripheral. Additionally, as the number of opinion dimensions increases, fewer clusters form, indicating that a broader range of opinions leads to fewer, larger groups. This study provides insights into the feedback loop between agent movements and opinion polarisation, providing insights on the mechanisms behind social segregation and the formation of echo chambers. Furthermore, these findings are crucial for understanding and addressing societal issues such as political polarisation, misinformation spread, and the dynamics of social cohesion. Our research contributes to the field of opinion dynamics by highlighting the significant role of physical space and movement in shaping collective opinions and social structures, offering a comprehensive framework for future studies on the interplay between mobility, opinion formation, and polarisation.

## Introduction

We have opinion on almost everything, and this conditions what we do and how we live, also in relation to others. Opinions are influenced by the individuals we come into contact with, and this contact is often influenced by geographical location where we live (Macy et al., 2003). Therefore, physical space plays a crucial role in the formation and exchange of opinions and the level of polarisation (Zhang et al., 2018; Small & Adler, 2019).

Opinions and beliefs shape the inner state of individuals, and these also determine people’s actions. When groups of individuals with shared beliefs gather together, these coalitions can become strong enough to function as clans and drive the spread of their ideas. Coupled with shared location, opinion convergence also generates collective actions (Centola, 2010; Hassanpour, 2014; Törnberg & Törnberg, 2017; Törnberg, 2018). When opinions are the driver for clique formation, these clusters may tip into social movements, protests or riots, like support for climate change initiatives (Smith et al., 2020; Winkelmann et al., 2022).

However, clustering with similar people, like people with similar opinion, is also a case of segregation (Schelling, 1971; Weisbuch et al., 2002), enhancing the risk of cultural differences (Axelrod, 1997; Centola et al., 2007) or the emergence of echo chambers and propagation of false information (Jamieson & Hardy, 2014; Williams et al., 2015). In echo chambers, opinions may become increasingly entrenched, leading to a lack of open-mindedness and unwillingness to consider alternative perspectives (DelVicario et al., 2016; Botte et al., 2022).

It is therefore important to increase our understating on how micro-level interactions among a population of mobile agents lead to opinion clusters in space and to what extent such clusters are polarised, internally and within society. As individuals interact and change their views, they might relocate to areas where others share their beliefs, leading to the formation of opinion-based clusters or segregated communities. For instance, individuals might relocate after political discussions, seeking communities with similar or differing views. Also, people might influence each other differently on various topics. Someone who is influential in political discussions might not have the same impact in cultural debates. The cumulative effect of interactions on multiple topics can lead to complex opinion dynamics and our model captures this complexity by considering multiple opinions and the varied interaction patterns among agents.

This paper addresses therefore the following questions: In what ways does the movement of individuals influence the dynamics of opinions? Does the formation of opinion clusters contribute to an increase in polarisation? To what degree does this clustering lead to a convergence of opinions within the entire system? Further, considering that individuals inherently hold multiple opinions simultaneously, how does this factor affect the formation of clusters? Are clusters formed by agents despite having divergent opinions on multiple matters? Or, are clusters exclusively formed among individuals with like-minded views?

This paper presents an extension to the Attraction-Repulsion Model (ARM) (Axelrod et al., 2021) and studies opinion dynamics and polarisation in a two dimensional (2D) space where agents’ neighbourhood size and geographic location constrains social interactions. Based on such geographic interactions, agents reduce or increase physical distance, endogenously forming opinion clusters. The paper contributes to the works on modelling opinion dynamics, studying the feedback loop between the movements of agents in a social space and polarisation of their opinion, highlighting its impact on the self-organisation of mobile agents into opinion clusters.

Opinion dynamics and agent movement result in preferential interactions among close-minded individuals, forming belief-based groups. Higher visibility leads to fewer, larger clusters, while, as for the original ARM, low tolerance drives high polarisation. Geographic constraints influence cluster positions in the 2D space: larger clusters centralise and those with opposite opinion distance themselves to avoid conflicts, while smaller clusters stay peripheral with less influence. As the number of opinion dimensions increases, fewer clusters form among agents, indicating that a broader range of opinions leads to fewer, larger groups.

### Models on Opinion Dynamic

Models on opinion dynamic aim to study the macro-level outcome in terms of opinion distribution, like convergence in contrast to polarisation (Levin et al., 2021), starting from assumptions on the micro-level actions determining social interactions. Polarisation, for instance, is often understood as a negative system outcome causing extreme behaviours and tensions in societies (Maertens et al., 2020; Vasconcelos et al., 2021). Studying opinion dynamics is therefore essential for regulators, as polarisation can hinder consensus, intensify the propagation of false information, and undermine effective governance, necessitating the use of models to explore opinion formation mechanisms and identify intervention opportunities.

Several models on opinion dynamics have been developed in the literature (Castellano et al., 2009; Xia et al., 2011; Sîrbu et al., 2017; Li & Xu, 2022), addressing the fundamental question on how opinions form and spread in a population of heterogenous individuals. Most of these models focus on examining the dynamics of opinions and polarisation among voters of political parties (Axelrod et al., 2021; Macy et al., 2021), with some evidence that such models can predict election outcomes and unexpected events (Galam & Cheon, 2020).

Models on opinion dynamics have also studied the climate change debate that has become highly polarised (Willaert et al., 2019). Results show that opinion formation impacts people’s perception of climate change as an urgent issue and the willingness to support measures aimed at mitigating it (Zanocco et al., 2019; Hazlett & Mildenberger, 2020; Motta, 2021; Mayer & Smith, 2023). Consequently, raised climate awareness drives social movements toward climate actions (Jamison, 2010; Pearse, 2016; McAdam, 2017; Drake & Henderson, 2022), as well as pro-environmental behaviour and lifestyle changes (van de Ven et al., 2018; Creutzig et al., 2022).

The literature of opinion dynamics has produced numerous models that incorporate diverse feature of social interactions, including the network structure in which agents (or nodes) exchange opinions (Palla et al., 2007; Vazquez et al., 2007; Vasconcelos et al., 2021) or the lattice space where neighbours impose constraints on interactions (Gracia-Lázaro et al., 2009; Pfau et al., 2013; Guo et al., 2015; Zhang et al., 2018). There is also a vast literature that models dynamic change depending on social norms and social learning (Galam & Jacobs, 2007; Centola et al., 2018; Kaaronen & Strelkovskii, 2020; Wiedermann et al., 2020). However, only a limited number of models have explored opinion dynamics in the unconstrained 2D space, which is the focus of this paper.

Starnini et al. (2016) study agents in a 2D space, that are influenced by neighbours and can reset their opinions. Movement is random, and heterogeneous neighbourhoods are fragile as agents avoid not-like-minded peers. Their focus is on mobility, homophily, and social influence in opinion dynamics, resulting in a meta-population structure where like-minded individuals naturally segregate. Levis et al. (2020) present a reference model with feedback between agents’ mobility and information spread. This impacts collective behaviour in physical and conceptual spaces. Mobility positively influencs information spread, leading to cooperative behaviours in physical space. Alraddadi et al. (2020) study how opinions and location co-evolve. Agents do not form explicit links but interact with local peers, and they decide to move based on attraction to similar opinions or repulsion from differing opinions. Increased mobility accelerates convergence, reduces local diversity, and allows multiple opinion clusters to coexist.

This paper aims to contribute to this literature on formation of opinion clusters and moving agents, differentiating from models of rewiring networks, model of moving agents in a lattice or model with random movements (Guo et al., 2015). We present a model that considers free-space movement, studying unconstrained opinion dynamics, opinion cluster formation and polarisation in a 2D space as result of social interaction and the emergent feedback loop effects.

This paper extends the original ARM (Axelrod et al., 2021), where both attractive and repulsive interactions drive opinion dynamics and polarisation, by introducing two main features (full model details in Materials and Methods). First agents are located in a 2D space. This implies that agents have both attractive and repulsive interactions based on their opinion and these interactions result in physical attraction or repulsion, meaning that an agent moves closer or away from the other interacting agent. Second, interactions between agents occur based on their geospatial distance, as one agent can only see and interact with another agent if the latter is in within a certain threshold space.

As agents’ visibility limits interactions, the model falls under the category of a bounded confidence model (BCM), where the one possible outcome is the formation of distinct clusters depending on the visibility threshold values (Weisbuch et al., 2002; Askitas, 2017; Alraddadi et al., 2020). However, attractive interactions can also cause agents to converge towards a single point in the 2D space (Takesue, 2023). Consequently, the model enables the study of the conditions under which ordered or disordered domains emerge.

Interactions between agents are not universal, but rather depend on their positions in a social space. Specifically, only agents within a certain proximity can interact. This type of interaction is similar to what is seen in other threshold models (Granovetter, 1978), where agents only interact and update their opinions if they fall within a certain level of confidence with each other. Such type of interactions may create clusters of like-minded individuals and can lead to polarisation in the network. But, since agents can have opinion on multiple matters, clusters may also be internally polarised (Sieber & Ziegler, 2019; Steinbacher & Steinbacher, 2019; Steinbacher et al., 2023).

This paper does not study how the network of agents evolves over time, nor the initial network topology of agents. It has been demonstrated that, when agents self-organise in clusters with repeated rewiring dynamics, the resulting network is a random network with not much structure. Similarly, the initial network topology (i.e., small words, or other) does not always influence the dynamics and results: as agents move as long as they interact, this dynamic generates a social network that turns into a random network with a Poisson degree distribution (Centola et al., 2007; Vazquez et al., 2007).

Our extended ARM considers agents that have no constraints in terms of network/social links, as their social connections are bounded only by their geographic range of visibility: the more they are able to see others the denser their social network in a given point in time. Visible agents, hence those to interact with, change every time an agent moves, changing its position and neighbourhood. This replicates the notion of *immediacy*, where social impact and influence of a person on another one depends on the physical distance (Latané, 1981; Nowak et al., 1990).

The model also expands previous opinion dynamic models run on unconstrained 2D space (Starnini et al., 2016; Levis et al., 2020; Alraddadi et al., 2020). Agent’s attraction to (or repulsion from) another agent depends simultaneously on their mutual opinion and geospatial location, rather than random movement. Further, our model considers agents having an opinion on multiple matters, impacting both overall system polarisation and clusters’ characteristics (i.e., size, intra-group polarisation).

Further, this paper follows the approach of several opinion dynamic models that assume a single activation at a time (Axelrod, 1997; Axelrod et al., 2021), despite real life interactions usually concern exchanges with multiple peers simultaneously. In the model, agent’s opinion is treated as continuous on a scale of 0 to 1, rather than discontinuous. This assumption reflects the nature of real social influence, where opinions change gradually without abrupt jumps from one opinion to another. Additionally, the experiments conducted in this paper do not investigate the initial distribution of opinions, but a check on result consistency is run. This is because it is commonly observed that populations tend to converge towards opinion consensus or polarisation regardless (Stokes et al., 2022).

By incorporating these new features into the original ARM, the aim of this study is to offer valuable insights into the role of opinion dynamics and spatial movements for opinion polarisation. Such social phenomena enable endogenous coordination among agents that, in turn, has the potential to influence social and collective actions. In an unconstrained space, opinion clusters can emerge where individuals collectively discuss and shape ideology, behaviour and lifestyle changes that can challenge those prevalent in the society or among dominant groups.

## The Model

### The Original ARM

The Attraction-Repulsion Model (ARM) was developed by Axelrod et al. (2021) to study opinion dynamics and ideological polarisation. In the model, opinion polarisation is driven by both attractive and repulsive interactions, and authors examine the conditions under which polarisation becomes a runaway process. Findings show that tolerance towards alternative viewpoints, coupled with a certain degree of exposure and responsiveness to those viewpoints, plays a significant role in limiting polarisation.

The model presented in this paper follows the dynamics of the ARM (Axelrod et al., 2021) and its two simple rules: the interaction and the attraction/repulsion rule.^1^ For simplicity, we first present the original ARM considering interactions among agents having an opinion on one matter only ($F=1$). Later in this section we also provide additional explanation on the model dynamic when agents interacts based on their opinion on multiple matters, $F>1$ (i.e., modelling agents with multiple opinion, also referred to opinion dimensional space higher than 1, or multiple ideological dimensions).

The first ARM rule considers each agent *i* ($i \in N$, where *N* is the total number of agents) having own opinion $Opn_i\in [0;1]$ on an issue. As common in opinion dynamic models, the model does one event at a time to avoid any effect of synchronous activation of multiple agents at the same time (Axelrod, 1997). This means that only one agent is activated at random every time step. Therefore, an agent *i* is selected and can interact with another agent *j* ($j\ne i$; $j\in N$), also selected at random. They interact with probability $\Psi _{i,j}$ (Eq. 1), that is function of the opinion distance $d_{i,j}$ between *i* and *j* (Eq. 2) and of the agents’ exposure to other points of view $E_i$. Probability $\Psi _{i,j}$ decreases with distance $d_{i,j}$, halving the distance of $E_i$. The higher $E_i$, the higher the probability an agent has to interact with other agents, either with those at an higher option distance or with those with similar opinion.

1 $$\begin{aligned} \Psi _{i,j}=(1/2)^{\frac{|d_{i,j}|}{E_i}} \quad \text {;} \quad \Psi _{i,j}\in [0;1] \text { , }E_i\in [0;1] \end{aligned}$$

2 $$\begin{aligned} d_{i,j}=Opn_i-Opn_j \quad \text {;} \quad d_{i,j}\in [-1;1] \end{aligned}$$

If agent *i* interacts with agent *j*, this interaction can result either in opinion attraction or repulsion. The second ARM rule (Eq. 3) in fact says that, if agent’s *j* opinion ($Opn_j$) is within agent’s *i* tolerance level $T_i\in [0;1]$, agent’s *i* new opinion ($Opn_{i}^{'}$) moves a fraction (responsiveness, $R_i\in [0;1]$) closer to agent’s *j* opinion. Otherwise, $Opn_{i}^{'}$ departs a fraction $R_i$ from $Opn_j$. In other words, an opinion distance between two agents lower then the tolerance level results in attractive interaction, otherwise an opinion distance higher than the tolerance level creates repulsion among the two agents. Formally:

3 $$\begin{aligned} Opn_{i}^{'} = \left\{ \begin{array}{ll} \text {Attraction}\, : Opn_{i} - R_i * d_{i,j} & \text {if } d_{i,j}\le T_i \\ \text {Repulsion} : Opn_{i} + R_i * d_{i,j} & \text {if } d_{i,j}> T_i \end{array}\right. \end{aligned}$$

Agents within the tolerance limit are more likely to interact, hence repulsion is less likely to occur than attraction, but it generates greater movement. Nonetheless, the likelihood of interaction and magnitude and direction of opinion change are uncorrelated. The parameter $T_i$ can also be interpreted as a proxy for contrarian behaviour. Agents with very low tolerance levels consistently reject opinions that differ even slightly from their own, triggering repulsive interactions that amplify both opinion and spatial distance. This effectively mimics contrarians, who systematically oppose opinions they encounter. Thus, the setup of the original ARM naturally captures contrarianism as an emergent behavior when tolerance levels approach zero.

In the ARM model, polarisation is measured as the variance of opinion in the population of agents, and, it can assume values between 0 and 0.25. No polarisation occurs when all opinions converge to a single point and variance of all opinion is 0 (e.g., when all agents’ opinion is at 0.5). Full polarisation occurs when two groups of the same size have the two extreme opinions, leading to variance 0.25 (e.g. where half of the agents has opinion 0 and the other half has opinion 1, opinion variance is 0.25).

#### The Extended ARM

The extended version of the ARM builds on these simple rules and dynamics and adds few important features. First, agents are located on a 2D space, and, this geographic position reduces possibility to interact. Second, an agent interacts with others, not only in relation to the opinion distance ($d_{ij}$) and exposure ($E_i$), but this interaction is function of an agent’s visibility, a proxy of the size of its neighbourhood. Third, agents move within the 2D space after each interaction. With this extension, the model analyses the effect of geography on opinion dynamics and polarisation and provides insights on how clusters are endogenously created based on people’s opinions. The formal details of the extensions to the ARM are elaborated below.^2^

Agents are located randomly on a grid, that is a Cartesian coordinate system. Each agent has a pair of numerical coordinates (*x*, *y*) that define their location into the Cartesian system, where $x\in [0;1]$ and $y\in [0;1]$. Each agent is randomly assigned with a parameter visibility ($V_i$) that quantifies the maximum surrounding area in which an agent *i* can interact with other agent *j*, if the latter is located within agent’s *i* neighbourhood. In other words, $V_i$ quantifies the max distance an agent is able to see other agents: the higher $V_i$ the higher the probability to interact with (e.g., see) neighbouring agents. The geographic distance (e.g., the Euclidean distance) between two agents ($z_{i,j}$ in Eq. 4) is computed based on their location (i.e., their Cartesian coordinates).

4 $$\begin{aligned} z_{i,j}=\sqrt{(x_i-x_j)^2+(y_i-y_j)^2} \quad \text {;} \quad z_{i,j}\in [0;\sqrt{2}] \end{aligned}$$

Opinion influence depends on each agent’s visibility (or neighbourhood size), meaning that agent’s opinion may be influenced only by other agents’ opinion if these agents are in the neighbourhood of this agent. Therefore, the probability to interact does not only depend on $\Psi _{i,j}$ (Eq. 3), but agent *i* interacts with agent *j* also subject to probability $\Phi _{i.j}$ (Eq. 5).

5 $$\begin{aligned} \Phi _{i,j}=1-{\frac{z_{i,j}}{V_i}} \quad \text {;} \quad \Phi _{i.j}\in [0;1] \text { , }V_i\in [0;\sqrt{2}] \end{aligned}$$

Probability $\Phi _{i.j}$ is a linear function that decreases with the increase of the ratio between the distance between agent *i* and agent *j* ($z_{i,j}$), and agent’s *i* visibility ($V_i$). In other words, the higher the distance the lower the probability to interact. This differs from the probability to interact based on opinion distance that follows Eq. 1. Of course, an agent takes into account only those agents that are within its visible space: hence only agents *j* for which $z_{i,j}\le V_i$ is true, otherwise $\Phi _{i,j}=0$.

When agent *i* interacts with agent *j*, its opinion adjusts following the attraction/repulsion rule of Eq. 3. Whether this interaction results in opinion attraction or repulsion, agent *i* moves towards or away from agent *j*, on a new position ($x_{i}^{'},y_{i}^{'}$) in the Cartesian space. Agents’ movements, and their new coordinates, are subject to Eqs. 6 and 7 below.

6 $$\begin{aligned} x_{i}^{'} = \left\{ \begin{array}{ll} \text {Attraction}\, : x_i-[(x_i-x_j)*M_i] \\ \text {Repulsion} : x_i+[(x_i-x_j)*M_i] \end{array}\right. \end{aligned}$$

7 $$\begin{aligned} y_{i}^{'} = \left\{ \begin{array}{ll} \text {Attraction}\, : y_i-[(y_i-y_j)*M_i] \\ \text {Repulsion} : y_i+[(y_i-y_j)*M_i] \end{array}\right. \end{aligned}$$

Equations 6 and 7 say that, after each interaction, agent *i* moves a fraction $M_i\in [0;1]$ towards or away from agent *j*. With maximum mobility, that is when $M_i=1$, in case of repulsion, agent *i* doubles the distance to agent *j*, otherwise, in case of attraction, agent *i* moves in the same position of agent *j*. When $M_i=0$, regardless the type of interaction, agent *i* does not change its position. When agents *i* and *j* are in the same location (hence same coordinates), their interaction-whether attractive or repulsive-affects their opinion but does not lead to movement. Given that their geographical distance is zero ($x_{i}-x_{j}=0$ and $y_{i}-y_{j}=0$), any interaction, regardless of the agents’ mobility, results in a movement of zero distance.

Movements are constrained within a specific Cartesian space, where the new coordinates for agents are restricted to values between 0 and 1. Essentially, this implies that when an agent reaches any of the four sides of the 2D space, it becomes unable to move away and can only be attracted towards the centre, if applicable. This limitation becomes even more restrictive when an agent reaches one of the four corners of the space. This can create artificial spatial clustering along the edges and corners, since these agents have fewer possible interaction and are less likely to disperse further. This effect may exaggerate polarisation in these areas, as agents trapped near the boundaries may form persistent clusters due to reduced exposure to others. Such boundary effects are inherent to spatial agent-based models with finite geographic space and should be kept in mind when interpreting clustering and polarisation outcomes. However, these effects can also be seen as a stylised representation of physical constraints in real-world geographic or social spaces, where physical or structural boundaries limit mobility and influence interaction patterns.

At the end of each simulation the number of established clusters is also calculated. The model simply looks at the number of agents located in the same position having the same opinion, with a margin $h=\pm 0.04$. The following algorithm is implemented. Select the first agent *i* and set its coordinates and opinion as those of the first cluster. Then, one by one, look to coordinates and opinions of the other agents. A second agent *j* is selected: if coordinates and opinion of agent *j* are 0.04 lower or higher than the first agent *i*, agent *j* belongs to the first cluster, otherwise a new cluster is established with coordinates and opinion of agent *j*. A third agent is then selected and it is allocated to one of the two established clusters if its coordinates and opinion are within the 0.04 upper and lower limits, otherwise another cluster is established. This process goes until all agents are allocated to one cluster.

This algorithm differentiates from more established ones, as for example the Density-Based Spatial Clustering of Applications with Noise (DBSCAN), formulated to identify clusters with arbitrary shapes and that uses the density-based concept of clusters (Ester et al., 1996). The extended ARM runs until stable (opinion and location) condition is reached, that is when all agents do not move after interactions and do not change opinions. Further, compared to the DBSCAN clustering algorithm that does not directly classify “loners” (clusters with less than $c=5$ agents), the clustering algorithm utilised in this model incorporates singletons ($c=1$), as the presence of size-one clusters is a crucial aspect to monitor. The existence of these individual clusters significantly influences both opinion clustering and the geospatial dynamics of movement.^3^

The extended ARM also test the *stubborn* behaviour of agents ($S_i\in [0;1]$) (Glass & Glass, 2021; Kareeva et al., 2024). This is the probability of agents’ being attracted to its original position even if it has moved already into a new location. $S_i$ has the same effect of $P_i$, that Axelrod et al. (2021) call *economic self-interest* and represents the probability to return closer to original opinion. Therefore, with fixed probability $P_i$ and $S_i$ respectively, a self-interested agent is attracted by its original opinion ($Opn_{i,t=0}$) and a stubborn agent is attracted by its original position ($x_{i,t=0}$ ; $y_{i,t=0}$) (Eqs. 8 and 9). Formally:

8 $$\begin{aligned} \text {Self-interested agent} {\left\{ \begin{array}{ll} Opn_{i}^{'} = Opn_{i} - [(Opn_{i}-Opn_{i,t=0}) * R_i] \\ \end{array}\right. } \end{aligned}$$

9 $$\begin{aligned} \text {Stubborn agent} {\left\{ \begin{array}{ll} x_{i}^{'}\, = x_i-[(x_{i}-x_{i,t=0})*M_i] \\ y_{i}^{'} = y_i-[(y_{i}-y_{i,t=0})*M_i] \end{array}\right. } \end{aligned}$$

*S* represents agents’ non-conforming behaviour, which is the opposite of the bandwagon effect, where people always conform to what the majority is doing. For example, individuals with stubborn behaviour may avoid popular locations in search of uniqueness, thus deviating from the norm. When an agent *i* is selected at random, with a probability *S* it decides to move closer to its initial location in the 2D space, without further interaction (hence it keeps current opinion). A value of $S=0$ indicates no probability of being stubborn, meaning that each interaction might results in either a repulsive or attractive movement. Conversely, $S=1$ implies completely stubborn behaviour: agents do not interact at all, thus being stationary and preserving their initial position and opinion.

#### Multiple Opinion Dimensions

The extended ARM also considers agents having multiple opinions ($F>1$). The model dynamic described above remains almost the same also in this situation. The main difference is that, agent *i* interacts with another agent *j* on only one dimension at a time. Each interaction relates to a specific opinion $f^{'}$ ($f^{'} \in F$, where *F* is the total number of opinions agents can have), and this can result in either attraction or repulsion. When the same two agents *i* and *j* interact on another opinion $f^{''}$ ($f^{''} \in F$), this can result in either attraction or repulsion, regardless their previous interaction. This means that two agents might agree on one opinion dimension and disagree on another one, determining therefore repetitive attractive and repulsive movements in the 2D space.

In case of multiple opinions, and following the original ARM, overall system polarisation is the sum of polarisation for all *F* dimensions (e.g., with $F=2$ the maximum polarisation is 0.5). For what concern the number of established clusters, this is calculated by looking at the number of agents located in the same position (with a $\pm 0.04$ margin), regardless the opinion they have. This means that clusters in an opinion dimensional space higher than 1 can be internally polarised, hence established by not-like-minded agents.

**Table 1:** Model initialisation

Parameters		Value
Total population of agents	*N*	100
Opinion dimensions	*F*	1
Initial opinion of agent *i*	$Opn_{i}$	$\mu =0.5$, $\sigma =0.2$
Tolerance	*T*	0.25
Responsiveness	*R*	0.25
Exposure	*E*	0.1
Self interested	*P*	0
Initial position of agent *i*	$x_{i}$	$x_{min}$=0, $x_{max}=1$
	$y_{i}$	$y_{min}$=0, $y_{max}=1$
Visibility	*V*	0.75
Mobility	*M*	0.25
Stubbornness	*S*	0

For this reason, the model calculates the polarisation for each cluster. For each of the *F* opinions within a cluster, the polarisation is determined as the variance of that particular opinion among the agents in the cluster. Subsequently, the total polarisation of a cluster is the sum of the polarisation for all *F* opinions. The overall polarisation across clusters is computed as the variance of the polarisation of each individual cluster, while the polarisation within clusters is computed as the average of the polarisation of each individual cluster.

## Results

In this section, a set of simulation experiments is presented, examining the results from various conditions governing opinion dynamics and the impact on polarisation and formation of clusters within a population of moving agents. The model initialises the parameters according to Table 1, and these are the default values unless stated otherwise.

The model simulates a population of 100 agents that are homogeneous in respect to tolerance (*T*), responsiveness (*R*), exposure (*E*), visibility (*V*), mobility (*M*), and levels of self interest (*P*) and stubborn behaviour (*S*). Agents interact on one opinion dimension (*F*=1) and are heterogenous for their initial opinion in such dimension. Opinion initialisation follow the original ARM, which is based on a normal distribution with given average ($\mu =0.5$) and standard deviation ($\sigma =0.2$).^4^ Agents’ initial position in the Cartesian coordinate system is randomly assigned with a pair of numerical coordinates (*x*, *y*) drawn from a uniform distribution, where $x\in [0;1]$ and $y\in [0;1]$.

### Opinion Dynamics: Polarisation and Cluster Formation

The original ARM suggests that tolerance (*T*) is the most critical factor for polarisation when a low level determines high polarisation, opposite to high level of tolerance that reduces polarisation.^5^ In this section, we consider the impact of moving agents on the level of polarisation and we also study the extent to which agents establish opinion clusters.

**Fig. 1:**
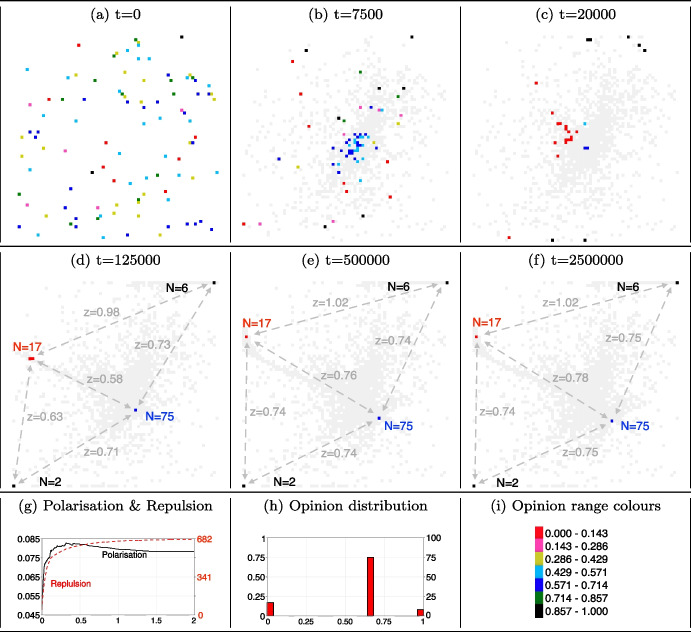
Opinion clusters, polarisation and opinion distribution. *Notes:* Plots (a-f) shows six time steps of the model simulation, where agents are located in the 2D space, and have different opinion detected by different colours. For graphical purposes, opinions are grouped in seven ranges as in plot (i). Plot (g) shows the level of polarisation (curve in black) and the cumulative number of repulsive interactions (dotted curve in red) for the entire simulation (2000000 time steps). Plot (h) show the opinion distribution after 2000000 time steps. Model parameters are initialised as in Table 1

Figure 1 shows the population of agents and their dynamic interactions in a 2D space, based on their opinions and movements. Initially, at $t=0$ (plot (a)), agents are randomly distributed across the Cartesian coordinate system, each having a distinct opinion. As interactions start, agents tend to converge towards the centre of the 2D space (plot (b)). This movement is influenced by their tolerance and visibility levels, which favour attractive interactions over repulsive ones as polarisation has not occurred yet. A majority of agents begin to form a cluster characterised by opinions within a middle-high range of values (ranging from 0.429 to 0.714, represented by the colours azure and blue).

As interactions continue, another opinion cluster begins to form in the same central location as the larger cluster (plot (c)). This new cluster is characterised by smaller size and overall extreme opinion, with low values ranging from 0 to 0.143 (represented in red in plot (c)). At the same time, a few more agents start to cluster in the top-right and bottom-left corners of the 2D space. These agents have opinions at the other extreme of the opinion spectrum, with high values ranging from 0.857 to 1 (represented in black in plot (c)). In the central location of the 2D space, two clusters with divergent opinions co-exist, enabling repulsive interactions. As shown in plot (d), the smaller cluster moves away from the larger one.

Four distinct opinion clusters emerge (plot (d)): the largest cluster at the centre (represented in blue with 75 members), two clusters in separate corners (in black, with 6 and 2 members respectively), and the last one (in red with 17 members) on the left-high side of the 2D space. Intra-cluster interactions occur between like-minded agents, implying that opinions become entrenched. This tend to reinforce existing viewpoints, thereby strengthening echo chambers.

Some inter-cluster interactions also occur, since geographic distance between the clusters remains below the visibility threshold ($V=0.75$). The distance between the central cluster and the cluster on the left-high side of the 2D space is 0.58. This proximity increases the probability of interactions among members of these two clusters more than interactions between agents in the central cluster and those in the corner clusters, given their greater distances (0.73 and 0.71, respectively). Tolerance level ($T=0.25$) favours repulsive interactions between agents belonging to distinct clusters.

At $t=12500$ (plot (d)), the 75 agents in the central cluster have an opinion value of 0.67 and those 17 in the left-high have opinion value of 0. When repulsive interactions occur between these two clusters, the left-high cluster tends to move away from the central cluster more than the central cluster moves away from the left-high cluster. The central cluster’s higher resistance to movement is due to its larger critical mass (75 agents vs 17). When an agent from the central cluster moves away due to repulsion from the left-high cluster, it has a higher probability of being attracted back by one of the agents in the central cluster, returning to its initial location. Conversely, when a left-high agent moves upper-left, the chances of it being attracted back to the original cluster are lower. These movements continue until the clusters reach a distance where interactions can no longer occur, as they end up locating outside each other’s visibility threshold. Their distance becomes 0.76 (plot (e)) and central agents have now opinion value of 0.70.

Agents located in the central cluster begin to interact exclusively with those situated in the corners of the 2D space (distances is still within the visibility threshold, both at 0.74). When a central agent interacts with an agent located in the top-right corner, the interaction tends to lead to repulsion, causing the central agent to move away from the centre. As a result, this agent becomes closer to agents in the opposite bottom-left corner. However, due to the tolerance level, another repulsion occurs, moving the agent back into the central cluster. Moreover, when a central agent deviates from the main cluster, there is a high probability that it will be once again attracted to other central members who still have similar opinions. As before, this tendency is reinforced by the critical mass of the central cluster, which increases the likelihood of interacting with like-minded agents.

Plot (f) illustrates the macro level outcome resulting from the interactions between central agents and the extreme agents. The largest cluster slowly moves away from the corner clusters, seeking an optimal distance that minimises repulsive interactions. In the end, the central cluster moves towards a safer location, avoiding interactions with contrarian agents, thereby ending social contacts and opinion exchanges.

The trend in polarisation shows a steep increase in the initial stage, reaching its maximum of 0.083, followed by a slow reduction in subsequent steps until it stabilises at a value of 0.078 (as shown in plot (g), black line). This fluctuation is attributed to the opinions of agents in the central cluster, since the other three clusters hold extreme opinions.

Initially, the central cluster has an opinion value of 0.67 (plot (d)), and interacts mostly with the left-high cluster, which has an opinion value of 0. This leads to repulsive interactions that cause the opinions of the central agents to deviate further, hence increasing from 0.67 to 0.70 (plot (e)). Once the distance between these two clusters prevents further interactions, the central cluster can still interact with the two corner clusters, which hold extreme opinion values of 1. These interactions are also repulsive, but in the opposite direction, causing opinions of central agents to revert back to the initial value of 0.67 (plot (f), summarised in plot (h)).

**Fig. 2:**
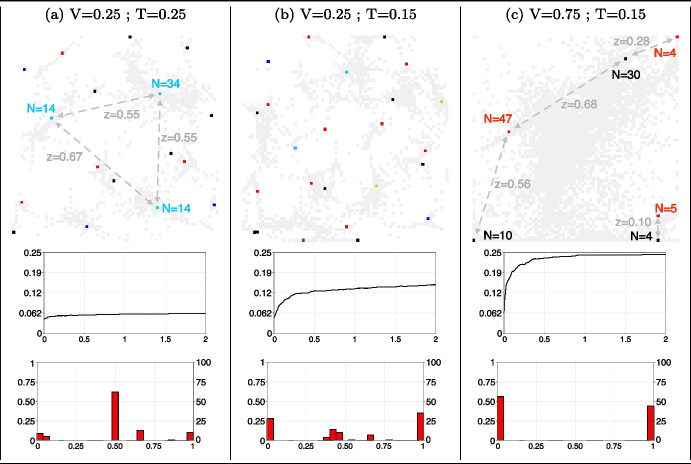
Opinion cluster and polarisation with varying levels of visibility and tolerance. *Notes:* Plots in each column show, from top to bottom, the final configuration of agents in the 2D space (opinion range colours as in plot (i) of Fig. 1), the polarisation trend, and the opinion distribution after 2,000,000 time steps. These are respectively for a population of agents having visibility and tolerance at the following levels: plots (a): $V=0.15$ and $T=0.15$, plots (b): $V=0.25$ and $T=0.15$, and plots (c) $V=0.75$ and $T=0.15$. Other model parameters are initialised as in Table 1

Plots in column (a) of Fig. 2 show the final configurations when agents have lower visibility ($V=0.25$) and maintain the same tolerance level ($T=0.25$). Lower visibility implies that agents have fewer interactions, as agents can be too far from each other to be seen mutually. This results in a larger number of established opinion clusters compared to when visibility was higher: 17 clusters in plot (a) of Fig. 2, compared to 4 in plot (f) of Fig. 1. The three largest clusters (with 34, 14, and 14 members respectively) tend to be located in the centre of the 2D space, as this position and their critical mass enable them to attract additional agents. Agents in smaller clusters (averaging size 2.7) have extreme opinions (either 0 or 1) and rarely interact with others. The polarisation level (central plot of column (a)) is low (0.061) but slightly higher than the case with higher visibility (0.078). This is because the majority of agents belonging to the three largest clusters have opinions of middle value (bottom plot in column (a)).

With the same low level of visibility ($V=0.25$) but a reduced tolerance ($T=0.15$), interacting agents establish an even larger number of opinion clusters in the 2D space (23, as shown in the upper plot of column (b) in Fig. 2). As expected, a lower tolerance results in higher polarisation, but not at its maximum value, as lower visibility still hinders interactions.

Full polarisation instead occur when visibility increases again ($V=0.75$) and tolerance is kept low ($T=0.15$), as shown in plots in column (c) in Fig. 2). Agents form 6 highly polarised clusters, at significant distances from each other. Polarisation and distance reduce interactions, therefore, even clusters with opposing opinions in closer proximity have very few repulsive interactions.

The final 2D configuration of column (c) is significantly influence by the most numerous clusters. The cluster in the top right corner (size 4 and opinion value 0) is less influential due to its smaller size compared to the nearest cluster on the left (size 30, opinion 1). This larger cluster receives a stronger repulsive effect when interacting with the largest cluster on its left (size 47, opinion 0) than with the one in the top-right corner. Therefore, this cluster size 30 locates closer to the smaller than to the bigger cluster (0.28 and 0.68 respective distances). Similarly, the biggest cluster (size 47) is confronted with both repulsive interactions from the bottom-left corner cluster (size 10, opinion 1) and the one size 30. The largest cluster positions itself between these two clusters of contrarians, but slightly closer to the smaller cluster (0.56 and 0.68, respective distances).

In summary, the movement of agents within a 2D space plays a crucial role in shaping opinion clusters. These clusters show internal opinion convergence (i.e., echo chambers of like-minded individuals), yet they remain polarised due to differing opinions within each cluster. Attractive interactions among agents lead to the formation of clusters, while repulsive interactions cause contrarian agents to move away from one another. Autonomously, clusters continue to distance themselves until they reach a safer location, effectively resolving conflicts: self-organising agents, including those with lower tolerance levels, naturally find a safe social distance to end conflicts. However, when multiple clusters coexist in the same space, and geographical constraints prevent them from locating in conflict-free zones, the critical mass of these clusters is crucial for overall stability. Larger clusters tend to occupy central positions in the 2D space, pushing smaller contrarian clusters away, further intensifying segregation of minority.

#### The Combined Effect of Visibility (*V*) and Tolerance (*T*)

Tolerance (*T*) and visibility (*V*) highly impact, respectively, polarisation and number of opinion clusters formed.^6^

**Fig. 3:**
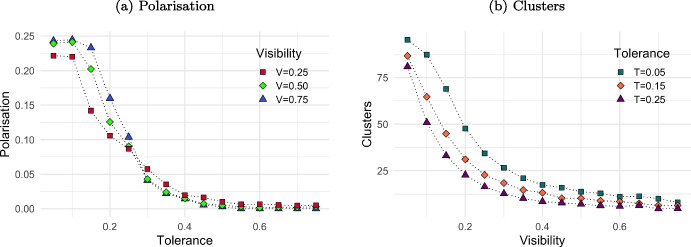
Combined effect of visibility (*V*) and tolerance (*T*) on polarisation and opinion clusters. *Notes:* Plot (a) shows the effect of tolerance (*T*) on polarisation by varying level of visibility ($V=0.25$, $V=0.50$ and $V=0.75$). Plot (b) shows the effect of visibility (*V*) on the number of clusters established by varying level of tolerance ($T=0.05$, $T=0.15$ and $T=0.25$). Values are averaged over 20 iterations after 2,000,000 steps. Other parameters assume values as in Table 1

Plot (a) in Fig. 3 highlights a significant decrease in polarisation when the tolerance is within the 0.15 to 0.25 range, leading to a convergence of opinions for subsequent values. Further, for each tolerance level within the 0 to 0.25 range, an increase in visibility corresponds to an increase in polarisation, reaching its peak at $V=0.75$.^7^ This trend reverses for tolerance values exceeding 0.3, where an increase in visibility results in reduced polarisation. Plot (b) in Fig. 3 shows a significant reduction in the number of opinion clusters as visibility exceeds 0.1 and continues until approximately 0.25. This trend results in a lower number of clusters at higher visibility levels. Within each visibility level, an increase in tolerance corresponds to an increase in the number of established opinion clusters, suggesting a potential linear relationship between tolerance and the number of clusters.

**Fig. 4:**
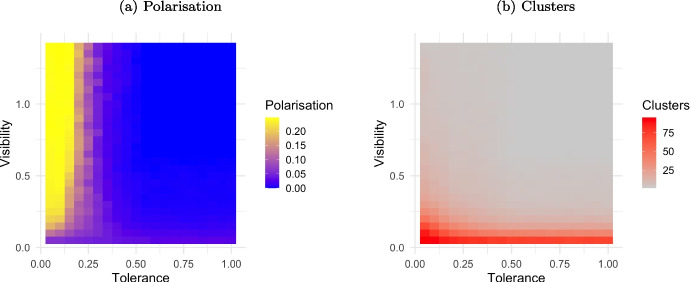
Polarisation and clustering with varying visibility (*V*) and tolerance (*T*). *Notes:* Plot in columns (a) and (b) show, respectively, polarisation and number of clusters based on different level of visibility (*V*) and tolerance (*T*). Values are averaged over 20 iterations for each pair after 2,000,000 steps. Values vary at 0.05 steps in the following ranges: $T\in [0.05,1]$ and $V\in [0;\sqrt{2}]$. Other parameters assume values as in Table 1

Referring to plot (a) in Fig. 4, when tolerance is low and visibility exceeds 0.3, extreme polarisation occurs (yellow cells). As tolerance increases beyond 0.25, and visibility surpasses 0.15, complete opinion convergence takes place (blue cells). With low visibility, moderate polarisation (purple cells) persists when tolerance is low. Looking to plot (b), when visibility is very low agents form numerous clusters (red cells), indicating a high degree of individualism. However, with higher visibility, approximately $V>0.15$, the number of clusters diminishes (grey cells), regardless of the tolerance values.

In agreement with the original ARM, low tolerance leads to extreme polarisation and high tolerance results in consensus. Moderate tolerance creates a balanced majority with some extremism, and this effect is amplified with lower visibility. A population of agent characterised by low visibility reduces social interactions leading to formation of several opinion clusters even with similar opinion. As long as agents increase visibility, they can interact with more people, easing clustering with like-mined peers in large groups. This results in dissemination of culture, with local convergence and, depending on the tolerance level, global polarisation.

Agents with very low tolerance levels ($T_i = 0.05$) systematically reject opinions that differ even slightly from their own, mimicking contrarian behavior. Contrarians therefore trigger repulsive interactions, increasing both opinion polarisation and the number of established clusters (see left side of both plot (a) and plot (b) in Fig. 4). The only case where this effect is mitigated is at very low visibility, when agents have limited opportunities to interact anyway.^8^

#### Mobility (*M*)

**Fig. 5:**
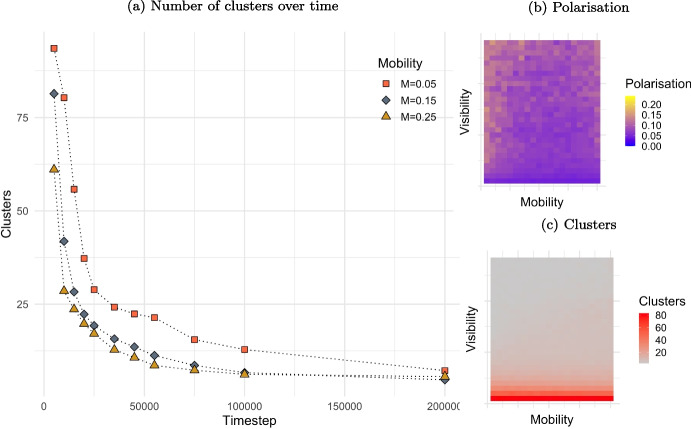
The effects of mobility (*M*) on speed of clustering, and its combined effect with visibility (*V*) on polarisation and clustering. *Notes:* Plot (a) shows the number of clusters established based on three different level of mobility: $M=0.05$, $M=0.15$ and $M=0.25$. Plot (b) and plot (c) show, respectively, polarisation and number of clusters based on different level of mobility (*M*) and visibility (*V*). Values vary at 0.05 steps in the following ranges: $M\in [0.05,1]$ and $V\in [0;\sqrt{2}]$. Values are averaged over 20 iterations for each pair after 2,000,000 steps. Other parameters assume values as in Table 1

Mobility impacts the speed at which stability is reached among agents, hence the speed at which clusters are established. Plot (a) in Fig. 5 shows that higher mobility results in rapid cluster formation and configuration stability. High mobility leads to fast relocation after interactions, forming echo-chambers directly (attractive interactions) or indirectly (repulsive interactions). Low mobility results in slow movements into the 2D space, requiring higher number of interactions to reach stability.

The effect of mobility on polarisation (plot (b)) and on the number of clusters (plot (c)) is less relevant as slow mobility lead to same outcomes, but after longer time. When visibility surpasses 0.15, agents tend to form only a small number of clusters, regardless of the level of mobility. This also leads to slightly higher polarisation on average, as described in previous section.

In a population of agents who quickly move toward or away from others based on whether they agree or disagree can rapidly form tight-knit groups. This quick grouping shortens the time they spend in disagreement. On the other hand, if individuals move slowly, it takes longer for groups to form and for conflict to be terminated.

#### Stubborn Behaviour (*S*)

**Fig. 6:**
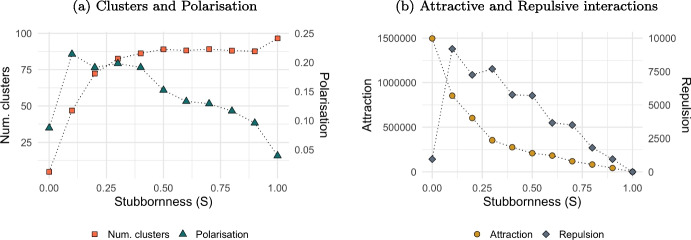
The effect of the level of agents’ stationary on polarisation of cluster formation. *Notes:* Plot show the level of polarisation (left vertical axis in blue) and number of clusters established (right vertical axis in orange) based on different level of stationary (*S*). Values are averaged over 20 iterations after 2,000,000 steps. Other parameters assume values as in Table 1

A very small probability of being stubborn leads to high polarisation and a low number of established opinion clusters. Polarisation decreases linearly with stubborn behaviour, resulting in no cluster formation (plot (a) of Fig. 6).

When agents are not stubborn ($S=0$), they tend to quickly form clusters with like-minded agents, creating a critical mass before distancing themselves from clusters of contrarians (see analysis in Fig. 1). Consequently, agents move to a specific location in the 2D space. A small probability of being stubborn creates tensions in this stable configuration: agents continue to form clusters with like-minded agents, but on rare occasions, an agent deviates from this location. So, agents deviating from mass conformity increase the risk of encounter contrarians leading to further opinion repulsion, thereby boosting polarisation (see plot (b)). This type of repulsion is different from the repulsion that occurs when $S=0$, as in this case, agents also move far away from each other, tending to move outside each other’s visibility threshold. In contrast, moving closer to the initial location due to stubborn behaviour does not take others into account and increases the chance of moving into the visibility range of an agent with an opposing opinion.

As the level of being stubborn increases, this lead agents to deviate very often from the mass, reducing interactions. Increasing the probability of being stubborn reduces the chance of agents forming opinion clusters with like-minded agents. Most of the time, when an agent is selected, it decides to remain closer to its initial location, reducing the number of both attractive and repulsive interactions. This gradually leads agents to keep their initial opinion and position, becoming fully stationary ($S=1$).

**Fig. 7:**
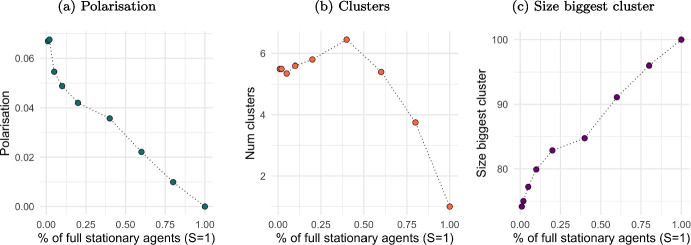
The effect of fully stationary agents on polarisation and opinion cluster. *Notes:* Figure shows the impact of fully stationary agents ($S=1$) as a share of the total population ($N=100$) on polarisation (plot (a)), on the number of clusters established (plot (b)) and on the size of the biggest cluster established (plot(c)). Values are averaged over 20 iterations after a 1,000,000 simulation steps. Other model parameters are initialised as in Table 1

Stationary agents may also play the role of social hubs, since they do not change opinion nor position and are therefore inflexible agents (Galam & Jacobs, 2007). Such agents can potentially attract others towards their own opinion and location, potentially leading to a single cluster of similar opinions. Instead, Fig. 7 indicates that stationary agents do not have such power to aggregate the population into a single location. Simulations are conducted with a certain proportion of agents remaining fully stationary from the start, meaning they assume an opinion of 0.5 and are positioned at the centre of the 2D space ($x=0.5$ and $y=0.5$). Polarisation (plot (a)) gradually reduces, but never reaches 0, as stationary agents are not able to drive the formation of one cluster only (plot (b)), despite it grows with the share of fully stationary agents (plot (c)).

This happens even if stationary agents have high visibility ($V=0.75$) and, being at the central location, they can see all other agents. However, this situation does not allow them to attract the entire population because some agents having extreme opinions (either very low or very high) remain outside stationary agents’ tolerance level ($T=0.25$), resulting in repulsive interactions. This occurs until a certain threshold of population share of stationary agents (about 40%). After this level, and when a high proportion of the population is stationary and occupies the central location from the start of the simulation run, polarisation gradually reduces, eventually disappearing into a single cluster with full uniformity.

#### Density (*N*) and Multiple Opinions (*F*)

Does population density in 2D space impact opinion dynamics? And what does it happen when agents interact on multiple opinions?^9^

**Fig. 8:**
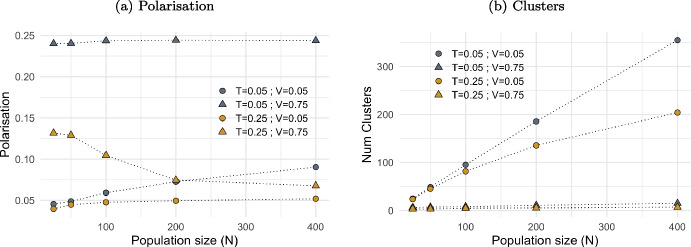
The effect of density on polarisation and opinion cluster. *Notes:* Figure shows the impact of population size ($N\in [25,400]$) on polarisation (plot (a)) and on the number of clusters established (plot (b)), at low and high level of visibility ($V=0.05$ and $V=0.75$) and tolerance ($T=0.05$ and $T=0.25$). Values are averaged over 20 iterations after a number simulation steps proportional to *N*, ensuring a maximum average of 20,000 activations per agent. Other model parameters are initialised as in Table 1

Plot (a) in Fig. 8 shows that, depending on the levels of tolerance (*T*) and visibility (*V*), an increase in the number of agents in the 2D space (*N*) can correspond to an increase in polarisation when both *T* and *V* are low. Conversely, it can lead to a decrease in polarisation when *T* and *V* are high. The number of clusters increases with population density, as shown in plot (b), but not proportionally. For instance, at low visibility and tolerance ($V=0.05$ and $T=0.05$) a doubling in population size corresponds to a lower degree of increase in terms of the number of clusters established. Instead, higher visibility ($V=0.75$) leads to very few clusters (on average between 3 and 15 in total).

**Fig. 9:**
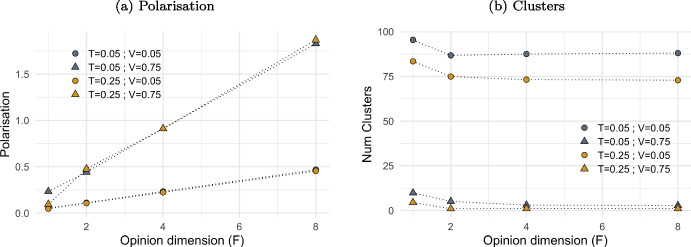
The effect of multiple opinions on polarisation and opinion cluster. *Notes:* Figure shows the impact of opinion dimension ($F\in [1,8]$) on polarisation (plot (a)) and on the number of clusters established (plot (b)), at low and high level of visibility ($V=0.05$ and $V=0.75$) and tolerance ($T=0.05$ and $T=0.25$). Polarisation is the sum of polarisation for all *F* dimensions, regardless clusters. Values are averaged over 20 iterations after a number simulation steps proportional to *F*, ensuring a maximum average of 10,000 activations per agent per dimension. Other model parameters are initialised as in Table 1

In a denser space, subject to a certain level of visibility, social interactions occur more frequently due to agents having a higher likelihood of being in close proximity. Density amplifies the effects of tolerance: intolerant individuals who interact more frequently due to closer proximity tend to increase polarisation and fail to form opinion clusters. Conversely, tolerant agents in an increasingly dense social space are inclined to reduce opinion differences, resulting in fewer but larger clusters.

Plot (a) in Fig. 9 shows that polarisation increases almost linearly with the size of the opinion dimension (*F*), but at varying rates depending on the levels of tolerance (*T*) and visibility (*V*). In case of multiple opinion dimensions, the overall polarisation within the agent population is the cumulative polarisation across each opinion.

As the number of opinion dimensions increases, the number of clusters formed by agents decreases: the greater the range of opinions agents interact upon, the fewer the clusters that emerge (plot (b)). Every activation, agents interact based on a single opinion dimension, independent of their position on other opinions. Consequently, since attractive interactions occur more frequently than repulsive ones (as shown in Fig. 17), agents generally move closer together rather than apart. Moreover, when agents converge at the same location due to a shared belief in one opinion, a repulsive interaction in another domain is unlikely to separate them significantly. In fact, once agents share the same exact location in the 2D space, a repulsive interaction may alter opinions but not result in movement, as their geographical distance is zero. Thus, any degree of mobility leads to no movement (see Eqs. 6 and 7).

**Fig. 10:**
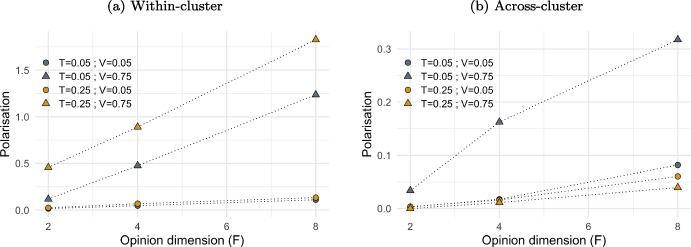
The effect of multiple opinions on polarisation within and across clusters. *Notes:* Figure shows the impact of opinion dimension ($F\in [2,8]$) on polarisation within clusters (plot (a)) and across clusters (plot (b)), at low and high level of visibility ($V=0.05$ and $V=0.75$) and tolerance ($T=0.05$ and $T=0.25$). Polarisation of a cluster is the sum of the polarisation for all *F* opinions. Polarisation within clusters is the average of the polarisation of each individual cluster. Polarisation across clusters is the variance of the polarisation of each individual cluster. Values are averaged over 20 iterations after a number simulation steps proportional to *F*, ensuring a maximum average of 10,000 activations per agent per dimension. Other model parameters are initialised as in Table 1

**Fig. 17:**
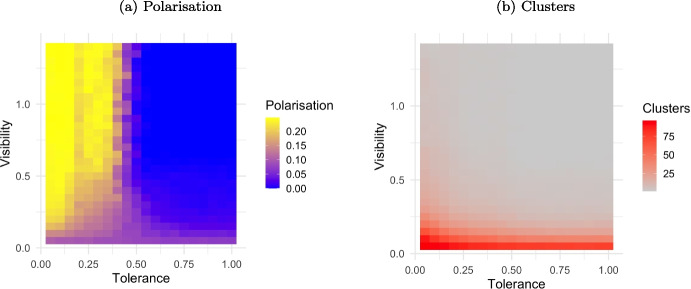
Polarisation and clustering with varying visibility (*V*) and tolerance (*T*) based on opinion initialisation following uniform distribution. *Notes:* Plot in columns (a) and (b) show, respectively, polarisation and number of clusters based on different level of visibility (*V*) and tolerance (*T*). Values are averaged over 20 iterations for each pair after 2,000,000 steps. Values vary at 0.05 steps in the following ranges: $T\in [0.05,1]$ and $V\in [0;\sqrt{2}]$. Opinion initialisation is drawn from a uniform distribution, where $Opn_{i}\in [0;1]$. Other parameters assume values as in Table 1

Figure 10 shows the effect of increasing the number of opinion dimensions (*F*) on two complementary measures of polarisation: within-cluster polarisation (plot (a)) and across-cluster polarisation (plot (b)). Both measures tend to increase at higher *F*, but the rate and extent of this increase depend on visibility (*V*) and tolerance (*T*). At very low visibility ($V = 0.05$), agents interact infrequently, which leads to the formation of many small clusters, often consisting of only one agent. In such cases, within-cluster polarisation remains low, as individual clusters are either very small or internally homogeneous.

In contrast, at higher visibility ($V = 0.75$), agents can interact with a much larger number of neighbours, resulting in the formation of fewer but larger clusters. Within these larger clusters, agents still maintain diverse opinions, which increases internal polarisation as *F* grows. Under these conditions of high visibility, low tolerance ($T = 0.05$) leads to high across-cluster polarisation, whereas this effect is much weaker when tolerance is higher ($T = 0.25$). With low tolerance, interactions are more likely to trigger repulsion rather than attraction, reinforcing differences between clusters, which become increasingly isolated and distant from each other. Instrad, higher tolerance facilitates broader opinion convergence, limiting the emergence of extreme polarisation across clusters.

## Conclusions

The paper has presented an extension of the Attraction-Repulsion Model (ARM) (Axelrod et al., 2021) where mobile agents are free to move into a 2D space. The goal of this paper was to study the interplay between agents’ movements and their opinion dynamics. The paper contributes to the effort to understand the dynamics of opinion and cluster formation, being patterns emerging as the collective result of interactions between heterogeneous and self-organising individuals.

The extended ARM simulates a system that is inherently chaotic and unpredictable and investigates whether it can reach stability through endogenous interactions among the agents, without any external intervention. As agents are able to move in the systems, their attraction to or repulsion from neighbours determines the formation of local community (clusters) of similar belief. Agents’ movements can cause changes in their opinion states over time, while opinion dynamics can also influence the geographic positions of agents and the opinions of nearby agents. This feedback loop between movement and opinion changes is the central component of the co-evolving dynamics of the extended ARM (i.e., system’s adaptive dynamics) and the main novelty of this paper.

Model outcomes show that the co-evolution of opinion dynamics and movements of agents mainly determines patterns of preferential interaction of close-minded individuals. When agents interact and move closer to each other, they tend to naturally form groups based on similar beliefs. Visibility (a proxy of the size of agents’ neighbourhood) significantly affects the number of clusters formed: higher (lower) visibility leads to fewer and larger (more and smaller) clusters. As for the original ARM, high (low) polarisation is driven by low (high) tolerance: opinions stop changing when the difference in opinions with people outside the group becomes larger than a certain tolerance limit. For instance, in residential neighbourhoods, people with similar socioeconomic backgrounds or cultural identities may cluster together, leading to segregated communities. Higher visibility of diversity (e.g., through community events or cultural exchanges) can promote integration and the formation of more integrated clusters, while low visibility may reinforce segregation in smaller homogeneous neighbourhoods.

Initial repulsive interactions (hence conflicts) occurring among dissonant opinion force agents to find stability far away each others. Therefore, opinion clusters naturally tend to locate at safe distance in order to avoid conflicts. However, when geographic boundaries force contrarians to form clusters that coexist in the same social space, their distance from each other is somehow proportional to the critical mass of the clusters. Bigger clusters position themselves at the centre of the space, maintaining a safe distance to avoid repulsive interactions with contrarians. Smaller clusters remains relegated to the sides and have less impact on larger ones due to their low critical mass, which limits their ability to shape the opinions of other clusters. Further, when a clusters is in between two contrarian clusters of different size, it located farther than largest cluster and closer to the smaller one. An example of such dynamics can be seen in a city with two rival teams, both having a passionate fan base. Clashes may occur during derbies or intense matches. After unfortunate violent confrontations, individuals involved often distance themselves to prevent further conflict, prompting event organisers to minimise opportunities for opposing fans to encounter each other. Furthermore, when one team’s supporters significantly outnumber the other one (for instance, simply by being more successful in a given season), they tend to gather in central locations, while smaller groups of the opposing team are relegated to peripheral locations.

Higher mobility, making agents moving quickly toward or away from others based on whether they agree or disagree, can lead to rapid formation of tight-knit groups, reducing time spent in conflict. Agents’ attachment to original location (the stubborn behaviour or stationary behaviour) creates tensions among those forming opinion clusters with like-minded agents because it increases conflicts. In extreme cases, attachment to original location is too strong that makes agents stationary, eliminating social interactions. However, in densely populated areas, the proximity increases social interactions with an effect on polarisation and on formation of opinion clusters. For example, in urban areas undergoing community development projects aimed at increasing social interactions, newcomers may quickly bond with others who share similar interests or values, forming close-knit communities. However, this can inadvertently exacerbate polarisation as new clusters may unintentionally exclude residents who do not fit the dominant demographic, leading to increased social tensions.

As group formation does not necessarily imply internal homogeneity, an opinion cluster can exhibit internal polarisation, where a wide spectrum of opinions exists within the same group. This diversity of thought is a natural aspect of human social dynamics. Nevertheless, intrinsic polarisation can sometimes escalate the risk of conflicts, disagreements and tensions within the group. For this reason, the model has also tested the effect of multiple opinion dimensions. As the number of opinion dimensions increases, the number of clusters formed by agents decreases: the broader the range of opinions agents interact upon, the fewer the clusters that emerge. In real-world situations, people often share the same physical space despite having differing opinions. For example, colleagues working together may share similar beliefs on work-related matters but disagree on personal issues. Similarly, sports fans gather at stadiums to support the same local team, even if they hold different political views. Therefore, having multiple opinions enables individuals to find common ground and come together despite differences in other areas, hence reducing segregation and conflicts.

### Limitations and Future Model Developments

This model can be applied to larger populations and is not limited to any specific organisational setting, such as external pressures or internal structures. The purpose of the model is not to make empirical predictions but to explore system-level dynamics resulting from assumptions about interpersonal interactions. Therefore, the model is highly abstract and needs to be empirically calibrated and tested. It is designed to investigate general properties of opinion polarisation and clustering in a 2D space and to capture lawful regularities that are broadly relevant.

The model can be expanded in a number of ways. Change in behaviour of some agents may generate cascade effects or consequences for the entire system, if, for instance, a policy intervention succeeds in shifting agents’ opinions. Policy interventions may be localised, meaning they impact only part of the population with the option to be impactful or not. Also, there could be more than one policy in place in the system, and these can either affect distinct groups in the population of agents. But such policies will also interact (both positively and negatively). Instead of policies, these external factors could also be technologies or any other elements in the system that shape agents’ behaviour, preferences, attitudes, or perceptions.

Addressing polarisation requires exploring interventions that promote dialogue and understanding between extreme opinion clusters. By simulating scenarios where external agents influence the opinions and locations of these clusters, the model can investigate whether such strategies foster convergence towards a shared belief and location.

An additional avenue for future research could consider introducing heterogeneity in agents’ visibility. In the current model, visibility is homogeneous across all agents. However, future extensions could explore how heterogeneous visibility influences polarisation and clustering. This would allow for the investigation of the emergence of opinion leaders, where agents with low visibility (hence few interactions) become central hubs, influencing others’ opinions and locations without themselves needing to move or adjust their own views.

Related to the above, another model development, it could interesting to incorporate alternative definitions of contrarian behavior. For instance, following (Iacomini & Vellucci, 2023), contrarians could be modeled as agents who deliberately oppose the views of highly visible opinion leaders. This type of structured contrarianism (where opposition is targeted specifically at central influencers) could capture additional real-world complexity, especially in context where certain figures (influencers, celebrities, or political leaders) polarise public opinion by triggering contrarian reactions.

## Figures and Tables

**Fig. 11:**
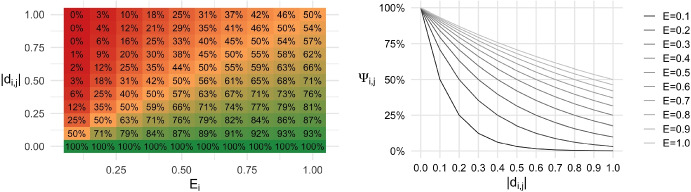
Agents’ interaction probability: $\Psi _{i,j}$, based on distance ($d_{i,j}$) and exposure ($E_i$)

**Fig. 12:**
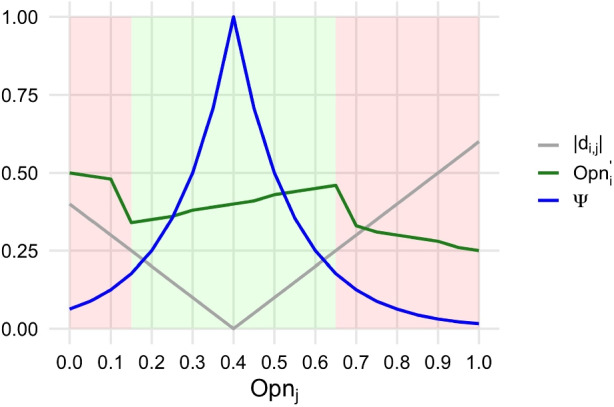
Example of agents’ interaction probability ($\Psi _{i,j}$) and opinion change based on attraction (area in green) or repulsion (area in red) rule

**Fig. 13:**
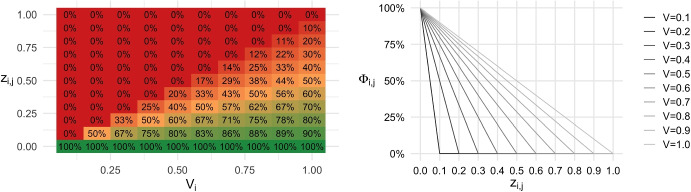
Agents’ interaction probability: $\Phi _{i,j}$, based on geographic distance ($z_{i,j}$) and visibility ($V_i$)

**Fig. 14:**
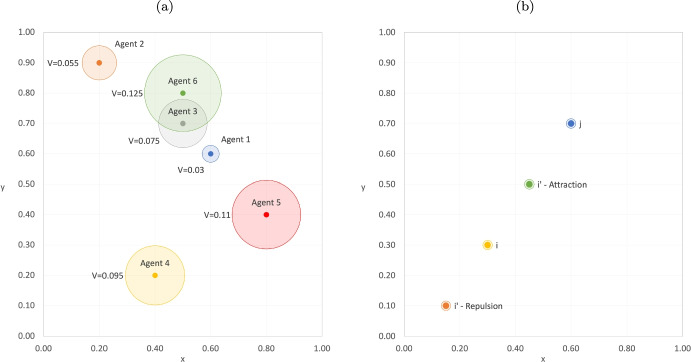
Example of agents located in a Cartesian plan. *Notes*: Plot (a) on the left shows the combined effect of agents position and visibility. Plot (b) on the right illustrates the effect of attractive and repulsion interaction among two agents

**Fig. 15:**
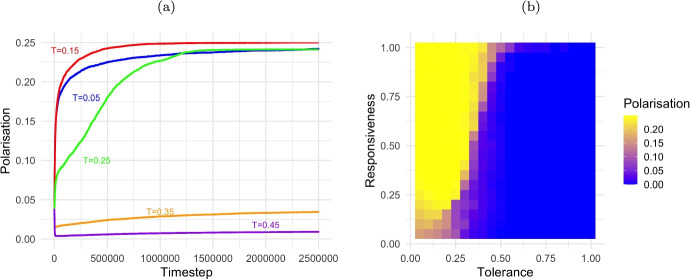
Replicate the original ARM. *Notes:* This two figures replicate key results of the original ARM (Axelrod et al., 2021): Plots (a) replicates Fig. 1 on the effects of tolerance (*T*); Plot (b) replicates Fig. 3 on the effects of responsiveness (*R*) as a function of tolerance (*T*). Source: authors’ analysis

**Fig. 16:**
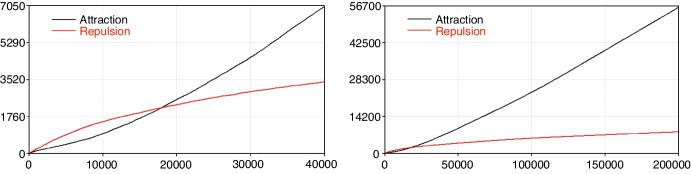
Number of cumulative attractive (in black) and repulsive (in red) interactions, during the initial 40000 time steps (on the left) and after 200000 time steps (on the right). This simulates the original ARM where the level of tolerance is very low: $T=0.05$. Source: authors’ analysis

**Fig. 18:**
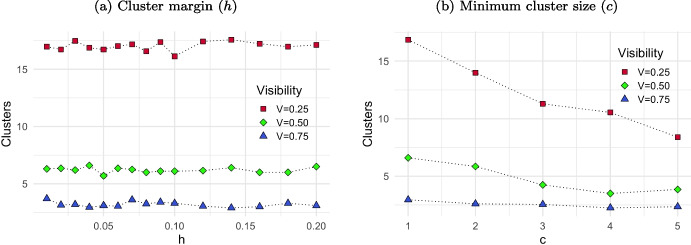
Impact of cluster margin (*h*) and minimum cluster size (*c*) on the number of opinion clusters. *Notes:* Plot (a) and Plot (b) illustrate the effect of cluster margin ($h\in [0.01, 0.2]$) and minimum cluster size ($c\in [1,5]$), respectively, on the number of clusters established under varying levels of visibility ($V=0.25$, $V=0.50$, and $V=0.75$). Values are averaged over 20 iterations after 2,000,000 steps. Other parameters are set according to Table 1

**Fig. 19:**
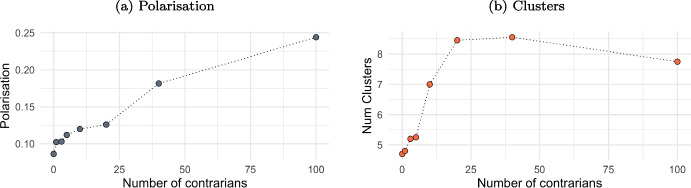
The effect of contrarians (agents with low tolerance) on polarisation and opinion clustering. *Notes:* Figure shows the impact of different number of contrarians ($T_{i}=0.05$) on polarisation (plot (a)) and on the number of established clusters (plot (b)). Values are averaged over 20 iterations after 2,000,000 simulation steps. All other model parameters are initialised as in Table 1

**Fig. 20:**
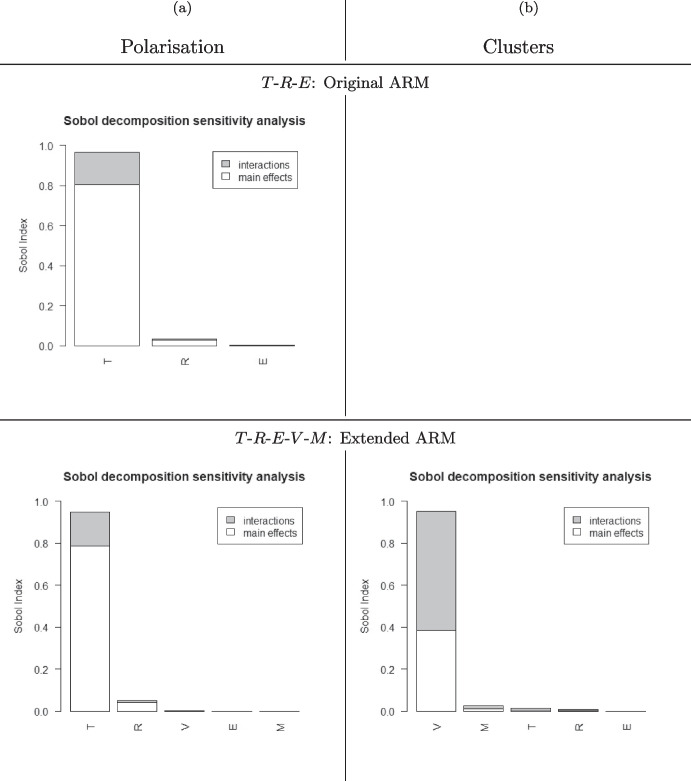
Sensitivity analysis: the Sobol decomposition. *Notes:* Charts show the effect of model parameters to the variance of the model outcomes: polarisation in plots in column (a) and the number of clusters established in column (b). Model parameters are initialised in the following range of values: $T\in [0.05,1]$, $R\in [0.05,1]$, $E\in [0.05,1]$, $V\in [0;\sqrt{2}$, $M\in [0.05,1]$. The direct effect (white area of bars) is the effect of each parameter on the output variance, and interaction (grey area of bars) is the parameter’s effect on the output variance based on interactions with other model parameters

## Appendix A: Annex

Additional insights related to both the original and the extended ARM are provided in this appendix. Specifically, Section A.1 elaborates on the rules governing the original ARM, while Section A.2 delves into the extended ARM. Further insights into the original ARM are presented in Section A.3. Section A.4 explores the model outcomes resulting from a uniform distribution of initial agents’ opinions, comparing them to the baseline initialisation using a normal distribution. Section A.5 presents the result of the sensitivity analysis on the clustering algorithm on the variations in the cluster margin and the minimum cluster size. Section A.6 explore heterogeneous tolerance, that is testing the impact of contrarian behavior among agents. Finally, Section A.7 offers an in-depth analysis of the importance of model parameters in relation to the variance of the model outputs.

### A.1: Rules Governing the Original ARM

Figure 11 shows how the probability to interact between two agents *i* and *j* ($\Psi _{i,j}$ in Eq. 1) varies in function of their opinion distance ($d_{i,j}$ in Eq. 2) and of the agents’ exposure to other points of view ($E_i$). $\Psi _{i,j}$ decreases with $d_{i,j}$, halving the distance of $E_i$. The higher $E_i$, the higher the probability an agent has to interact with other agents, either with those at an higher option distance or with those with similar opinion.

Figure 12 shows the dynamics of the attraction/repulsion rule via an illustrative example. We assume an agent *i* with $Opn_i=0.4$, $T_i=0.25$, $R_i=0.25$ and $E_i=0.10$. The plot shows the probability ($\Psi _{i,j}$, in blue) to interacts with agents with different opinions ($Opn_j\in [0;1]$) and the resulting new agent’s *i* opinion ($Opn_{i}^{'}$, in green) in case of interaction, and in relation to the opinion distance ($|d_{i,j}|$). Both $\Psi _{i,j}$ and $Opn_{i}^{'}$ are then represented in the plot on the right. Starting from 0.4 in x-axis, $\Psi _{i,j}$ decreases, both sides, as $Opn_j$ deviates from $Opn_i$: there is 100% chance to interact with another agent with same opinion, while this probability reduces to 50% if $Opn_j$ is either 0.30 or 0.50. When $Opn_j\in [0.15;0.65]$, $Opn_i$ adjusts, getting closer to $Opn_j$ (see curve in green): $Opn_i\in [0.34;0.46]$. In all the other cases, $Opn_i$ gets far away from $Opn_j$, because of repulsion between the two agents.

### A.2: Key Features of the Extended ARM

Figure 13 shows how probability $\Phi _{i.j}$ decreases linearly with the increase of the ratio between the distance between two agents ($z_{i,j}$), and agent’s *i* visibility ($V_i$): the higher the distance the lower the probability to interact.

Figure 14 plot (a) provides an illustrative example of agents located in a 2D space and how their level of visibility impacts interactions. Let us consider Agent-6 ($x_6=0.5, y_6=0.8$), with $V_6=0.125$, and Agent-3 ($x_3=0.5, y_3=0.7$). Based on Eq. 4, $z_{3,6}=0.1$ and $z_{3,6}<V_6$, implying that Agent-3 is in Agent-6’s visible area. Therefore Agent-6 can eventually interact with Agent-3. The opposite would not be valid, since Agent-3’s area is smaller and cannot see Agent-6 ($z_{6,3}>V_3$).

Figure 14 plot (b) provides an illustrative example on how agent *i* ($x_i=0.3$; $y_i=0.3$; $V_i=0.8$; $M_i=0.5$) moves after interaction with agent *j* ($x_i=0.6$; $y_i=0.7$). These agents have distance $z_{ij}=0.5$ (Eq. 4) and $\Phi _{i,j}=0.38$ (Eq.5). If their interaction results into attraction, agent *i* moves halfway closer to agent *j* ($x_{i}^{'} =0.45$; $y_{i}^{'} =0.50$), otherwise, in case of repulsion, agent *i* increases by 50% its distance to agent *j* ($x_{i}^{'} =0.15$; $y_{i}^{'} =0.10$).

### A.3: Replicating the Original ARM

The main result of the original ARM is that tolerance (*T*) is the most critical factor for polarisation among the parameters studied Fig. 15. A low level of tolerance in the population determines lower probability to interact, making repulsive interactions predominant and leading the population to stabilise in a very polarised environment. As soon as tolerance increases the population experience more attractive interactions, and polarisation reduces eventually disappearing with all agents converging to a single opinion. Axelrod et al. (2021) discuss crucial dynamics of the model and present how the effect of tolerance is in part mitigated by responsiveness (*R*) and exposure (*E*).

A more detailed analysis of the original ARM shows that repulsion only occurs during the first time steps of the simulation. Over the remaining steps of the model agents mostly have attractive interactions (see Fig. 16). In fact, at the very beginning of the simulation, given the initial distribution of agents’ opinion (that is modelled as a Gaussian distribution with average 0.5 and standard deviation 0.2), an agent has almost the same probability to interact with any of the other agents in the population. That is, there is higher chance to interact with another agent but resulting in repulsion. As shown in Fig. 16, with very low tolerance ($T=0.05$), during the initial interactions agents mostly experience repulsion. However, when opinion distance stabilises on average values, agents start to attract each other, determining a continuous grow on the number of attractive interaction among like-minded agents while the number of repulsive interactions stabilises. This makes opinions becoming increasingly entrenched leading to high polarisation.

### A.4: Testing Different Opinion Distribution

This section demonstrates that the initial opinion distribution among agents does not affect the final outcome. Due to the length of the simulations and the adaptive dynamics of the agents interacting within the model, numerous interactions lead to similar stable configurations (either convergent or polarised) regardless of the initial opinion distribution (Stokes et al., 2022). Figure 4 has shown the result of opinion initialisation using a normal distribution with a given mean ($\mu =0.5$) and standard deviation ($\sigma =0.2$). In this section, agents’ initial opinions are drawn from a uniform distribution, where $Opn_{i}\in [0,1]$. Figure 17 highlights almost overlapping outcomes with those shown in Fig. 4, confirming that, in the long run, the initial opinion distribution does not affect polarisation nor the number of opinion clusters established.

### A.5: Sensitivity Analysis on the Clustering Algorithm

Figure 18 shows the sensitivity of the clustering algorithm to variations in the cluster margin (*h*) (plot (a)) and the minimum cluster size (*c*) (plot (b)) across different levels of visibility. The cluster margin (*h*) defines the upper and lower bounds within which agents are considered part of the same cluster based on their opinion and spatial position. The parameter *c* sets the minimum number of agents required for a cluster to be counted, meaning that clusters with fewer than *c* agents are excluded from the count.

Analysing the model’s response to variations in *h* tests the robustness of the chosen default value ($\pm 0.04$) for clustering. Instead, evaluating the effect of *c* allows a comparative assessment of the clustering algorithm used in this study relative to the DBSCAN algorithm, which does not classify clusters smaller than a predefined size. However, it is important to note that DBSCAN operates based on core point and reachability degree, whereas the clustering algorithm used in this model considers only spatial proximity and opinion similarity.

Plot (a) in Fig. 18 shows that variations in the cluster margin (*h*) have a negligible impact on the number of clusters established. At each visibility level, the sensitivity analysis shows no significant change across the range $h\in [0.01, 0.2]$. Since simulations run until a stable configuration is reached and clusters naturally adjust their distances to minimize conflict, the impact of *h* is minimal, confirming the model’s robustness to variations in *h*. The default setting ($h=0.04$) is much smaller than the visibility threshold ($V=0.25$, as per Table 1), which explains its minimal impact. If $V \le h$, a significant reduction in the number of clusters would be expected, as distant agents that do not interact would be incorrectly grouped into the same cluster by the algorithm.

Plot (b) in Fig. 18 highlights the effect of the minimum cluster size (*c*) on the number of clusters. As *c* increases, fewer clusters are counted, with the impact being most pronounced at lower visibility levels. When visibility is low, agents have fewer opportunities to interact, leading to the formation of many small clusters. If *c* is increased, many of these small clusters are excluded from the count, significantly reducing the total number of clusters detected. In contrast, at higher visibility levels, agents have more interaction opportunities, forming fewer but larger clusters. Consequently, increasing *c* has a less effect in these cases with high visibility level.

### A.6: Contrarianism: Exploring Heterogeneous Tolerance

Following the formulation of the original ARM, our model is initially run under the assumption that all agents share the same tolerance level, and we test the model under different (homogeneous) tolerance levels across the population. However, the parameter $T_i$ (tolerance) can also be interpreted as a proxy for contrarian behavior, that is agents systematically opposing the opinions they encounter. In this section, we relax the homogeneity assumption and explore the case of heterogeneous tolerance, where only a fraction of the population acts as contrarians.

Following the parameter setup in Table 1, all agents are first initialised with the same tolerance level, $T_{i}=0.25$. Next, we randomly set a fraction of the population to be contrarians, assigning them the lowest tolerance level ($T_{i}=0.05$). This creates a mixed population where some agents are conformists (high tolerance) and others are contrarians (low tolerance). By gradually increasing the proportion of contrarians, we assess how increasing contrarianism impacts polarisation and clustering dynamics.

Figure 19 shows the results, with plot (a) showing polarisation and plot (b) the number of established clusters. When no agents are contrarians (fully homogeneous tolerance at $T_{i}=0.25$), the system converges toward high consensus (low polarisation) and few clusters, consistent with plot (f) in Fig. 1. As the proportion of contrarians increases, both polarisation and the number of clusters increase. When the share of contrarians exceeds approximately 25%, the system rapidly shifts toward high polarisation and elevated cluster formation. This result aligns with dynamics observed in other threshold models, where 25% often defines a critical tipping point for minority influence.

### A.7: The Sobol Decomposition Analysis

As is common in ABM literature (Dosi et al., 2018; Pasimeni & Ciarli, 2025), this section includes the Sobol decomposition analysis conducted on all model configurations resulting from different parameter combinations. The outcomes of these analyses are summarised in Fig. 20.

The Sobol decomposition is a method used for global sensitivity analysis of model parameters (Saltelli et al., 2000; Saltelli & Annoni, 2010). The Sobol analysis does not explain parameter variability, but it does provide an estimate of how much each parameter contributes to the variance in the model output. The decomposition distinguishes between the first-order index, which measures the individual effect of each parameter on the output variance (e.g., the direct effects), and the total-order index, which considers the parameter’s effect on the output variance, taking into account interactions with all other parameters (e.g., the interaction effects). The sum of the total-order indexes (e.g., direct plus interaction effect) can exceed 1 because mutual interactions are calculated twice for each parameter.

Referring to ARM, the Sobol decomposition quantifies the direct and interaction effects of each parameter on the variance of two main outputs: polarisation and number of clusters. The analysis considers the entire range of values for each parameter, from minimum to maximum (specifically: $T\in [0.05,1]$, $R\in [0.05,1]$, $E\in [0.05,1]$, $V\in [0;\sqrt{2}]$, $M\in [0.05,1]$). In the model, *P* and *S* are introduced explicitly to limit both opinion change and movement. Therefore, they are not included in the Sobol analysis since they have clear impact on level of polarisation and number of opinion clusters.

The initial model configuration (*T*-*R*-*E*) replicates the original ARM. In this case, tolerance (*T*) is the sole parameter directly impacting the variability of polarisation (direct effect equal to 0.80 and interaction effect lower around 0.20). The subsequent configuration (*T*-*R*-*E*-*V*) explores the extended ARM, focusing also on the number of clusters established by agents. The Sobol analysis reveals that polarisation variability is predominantly influenced by the direct impact of agents’ tolerance (*T*: direct effect equal about to 0.80 and interaction effect lower than 0.20). In contrast, the variability of the number of clusters depends largely on agents’ visibility (*V*) and on its interaction with other parameters (direct effect equal to about 0.40 and interaction effect lower than 0.60).

As observed in the original ARM study, tolerance plays a guiding role in polarisation. Lower levels of tolerance result in increased polarisation, while higher levels of tolerance lead to reduced polarisation. This dynamic remains consistent in the extended ARM. However, in the extended model, visibility governs the formation of clusters. Low visibility corresponds to a smaller number of clusters, whereas high visibility results in a larger number of clusters. Nonetheless, within these clusters, agents may exhibit varying levels of polarisation, indicating that the effect of tolerance remains unchanged compared to the original ARM.

Responsiveness (*R*) and mobility (*M*) do not have significant influence as they primarily slow down the mutual adaptation of agents, eventually reaching a stable state in the long run. On the other hand, the presence of stationary agents (*S*) has a clear impact on the number of clusters. These agents, attracted by their initial location, diminish the opportunity for clustering. Additionally, when these stationary agents are self-interested (*P*), meaning they are drawn towards their initial opinion and position, they further constrain polarisation and the formation of clusters.

## Footnotes

1 For more insights relative these rules governing the original ARM please refer to Annex A.1

2 For more details of the extended ARM please refer to Annex A.2.

3 In Annex A.5 we test robustness and sensitivity of the model to variations of both the cluster margin (*h*) and the “loners” cluster (*c*).

4 A random distribution of opinion is also tested, showing similar outcome. See Annex A.4 for more details.

5 For key findings and more insights of the original ARM, please refer to Annex A.3.

6 Please refer to Annex A.7 for more details on the importance of these model parameters in relation to the the variance of the model outputs.

7 For instance, at $T=0.25$ polarisation increases from 0.10 at $V=0.25$ to 0.12 and 0.16 at $V=0.50$ and $V=0.75$ respectively.

8 In Annex A.6 we incorporate contrarians into the model, hence exploring the effect of heterogeneous tolerance among agents.

9 Results of the experiments run in this section are determined by simulation steps that are not constant. For instance, with 100 agents and 2,000,000 steps, the model allows an average of a maximum of 20,000 activations per agent, which may result in opinion interaction and movement (only some activations lead to attractive or repulsive interactions, as both opinion and geographical distance decrease the probability of agent interaction (see Eqs. 1 and 5)). Therefore, to ensure model configurations with different population sizes (*N*) are comparable, an average maximum of 20,000 activations per agent is guaranteed. For example, with $N=50$, 1,000,000 steps are executed, while with $N=400$, 8,000,000 simulation steps are run. Similarly, in case of multiple opinion, an average maximum of 10,000 activations per agent and per opinion dimension is fixed, meaning that with $F=1$ and $F=8$, 1,000,000 and 8,000,000 simulation steps are run respectively.
